# An ultra-short screening version of the Recalled Parental Rearing Behavior questionnaire (FEE-US) and its factor structure in a representative German sample

**DOI:** 10.1186/1471-2288-12-169

**Published:** 2012-11-07

**Authors:** Katja Petrowski, Sören Paul, Markus Zenger, Elmar Brähler

**Affiliations:** 1 University of Leipzig, Department of Medical Psychology and Medical Sociology, Philipp-Rosenthal-Strasse 55, 04103 Leipzig, Germany; 2 Technische Universität Dresden, Institute of Clinical Psychology and Psychotherapy, Chemnitzer Str. 46, 01187 Dresden, Germany; 3 University of Leipzig, Department of Medical Psychology and Medical Sociology, Philipp-Rosenthal-Strasse 55, 04103 Leipzig, Germany; 4 University of Leipzig, Department of Medical Psychology and Medical Sociology, Philipp-Rosenthal-Strasse 55, 04103 Leipzig, Germany

**Keywords:** Rearing behavior, Screening instrument, Short form, Representative survey, Factor analysis

## Abstract

**Background:**

The Recalled Parental Rearing Behavior questionnaire (FEE, [1,2]) assesses perceived parental rearing behavior separately for each parent. An ultra-short screening version (FEE-US) with the same three scales each for the mother and the father is reported and factor-analytically validated.

**Methods:**

*N* = 4,640 subjects aged 14 to 92 (*M* = 48.4 years) were selected by the random-route sampling method. The ultra-short questionnaire version was derived from the long version through item and factor analyses. In a confirmatory factor analysis framework, the hypothesized three-factorial structure was fitted to the empirical data and tested for measurement invariance, differential item functioning, item discriminability, and convergent and discriminant factorial validity. Effects of gender or age were assessed using MANOVAs.

**Results:**

The a-priori hypothesized model resulted in mostly adequate overall fit. Neither gender nor age group yielded considerable effects on the factor structure, but had small effects on means of raw score sums. Factorial validities could be confirmed. Scale sums are well-suited to rank respondents along the respective latent dimension.

**Conclusion:**

The structure of the long version with the factors Rejection & Punishment, Emotional Warmth, and Control & Overprotection could be replicated for both father and mother items in the ultra-short screening version using confirmatory factor analyses. These results indicate that the ultra-short screening version is a time-saving and promising screening instrument for research settings and in individual counseling. However, the shortened scales do not necessarily represent the full spectrum covered by the full-scale dimensions.

## Background

Perceived parental rearing behaviour has an impact on child development [3,4]. Most empirical results were obtained in retrospective studies using two questionnaires: First, the Parental Bonding Instrument (PBI [5]) with its clinical version, the Measure of Parenting Style (MOPS [6]) and second the Egna Minnen Beträffande Uppfostran (EMBU, Own Memories of Child Rearing Experiences [7]). The EMBU [7] is a standardized questionnaire assessing four interrelated, factor-analytically derived dimensions of recalled parental rearing behavior for each parent [7,8]: Rejection, Emotional Warmth, Overprotection, and Favouring Subject. However, only the first three scales could be replicated in multinational and multicultural studies [9]. The EMBU long version has shown acceptable internal consistency, displaying Cronbach’s α ranging from .82 to .93 [10]. A short version with 22 items was derived through factor analysis and selecting the items with the highest squared loading (item reliability) on the scale [9] with seven items for Rejection, six items for Warmth, and nine items for Protection. Scales produced internal reliabilities of Cronbach’s α = .72 to .85 in student samples from Europe and Central America [9]. Norm values are available [2].

The German short version of the original EMBU, the Fragebogen zum erinnerten elterlichen Erziehungsverhalten (FEE, Recalled Parental Rearing Behavior [1,2]), comprises those eight translated EMBU items per scale that had the highest factor loadings in factor analysis. The FEE consists out of 24 items for each parent and is different from the English EMBU and short-EMBU [9,11] by content and scale composition, however, all three instruments rely on the same underlying concepts and result in the same three scales. For the FEE, they are defined as follows [1,2]: (1) Paternal/maternal Rejection and Punishment assesses overly strict, discerning parental behavior and rejections which were perceived to be inappropriate by the child. (2) Paternal/maternal Emotional Warmth assesses affectionate, supportive, praising behavior without implying unnecessary interferences by the respective parent. (3) Paternal/maternal Control and Overprotection assesses parental behavior which was perceived as overly thoughtful, blaming, interfering, and constricting, thus reflecting a distinct orientation on effort and high expectations by the respective parent. Psychometric properties as well as the factorial structure of the FEE short version with 24 items had been specified in a representative sample [12]. The FEE has already been implemented in different studies examining the validity of perceived parental rearing behavior [1,2,13-16], e.g. to examine the congruence of the recalled of the perceived parental rearing behaviour among siblings [13]. Furthermore, relationship characteristics as well as attachment patterns were successfully matched to the perceived parental rearing in clinical samples [14-16].

Recent development of psychometrics is more and more directed towards shorter instrument and screening questionnaires. Screening instruments were developed for the implementation in the prevention as well as the general medicine field to assess risk factors for or symptoms of psychopathologies, and enable rapid and targeted interventions (e.g. [17-19]). To screen for vulnerability factors in prevention and counselling studies as well as to identify risk factors early on, very short screening questionnaires are wanted. Besides, for the FEE only two to three items per scales showed acceptable factor loadings in the long version [2]. Hence, an ultra-short screening version (FEE-US) with two items for each of the three scales was developed using the items with acceptable selectivity. The correlation with the total value had to be r > 0.20 and positive so that the items cannot be misunderstood. In addition, the selected items had to show the highest factor loadings in previous representative studies [2]. As in the long version, the questionnaire consists of three scales for the father (with 6 items total) and three scales for the mother (with 6 items total) which are similar.

The factorial structure of this new screening instrument has not yet been evaluated. Therefore, the first objective of this study was to test whether the FEE factor structure can be replicated by the FEE-US by means of confirmatory factor analyses (CFA) using a representative, cross-sectional sample. The second objective was to test the sociodemographic specificity of the FEE-US scales, i.e. testing for differential item functioning and for differences in means of raw score sums. Results based on the English version showed that men may state more rejection in parental rearing behavior than women. Hereby, older subjects might idealize the parental rearing behavior more than the younger subjects do [8].

## Methods

### Sample

In 2006, the USUMA (Unabhängiger Service für Umfragen, Methoden und Analysen) Berlin Polling Institute selected households and participants by random-route sampling [20]. Sixty-two percent of all contacted persons filled out the questionnaire. Of these, only the final sample of *N* = 4,640 native German speakers who had grown up in a dual-parent household and who had completed the FEE questionnaire was examined (cf. Table 1). Using information from the Federal Statistical Office, the final sample was approved to be truly representative for the German residential population of 2006. All participants volunteered and received a data protection declaration in agreement with the Helsinki Declaration. The study was approved according to the ethical guidelines of the “German Professional Institutions for Social Research” (Arbeitskreis Deutscher Markt- und Sozialforschungsinstitute, Arbeitsgemeinschaft Sozialwissenschaftlicher Institute, Berufsverband Deutscher Markt- und Sozialforscher).

**Table 1:** Sample characteristics

		*** N***	**%**
**Gender**	male	2,128	45.9
	female	2,512	54.1
**Age** (years)	mean	48.4	
	standard deviation	17.96	
	range	14 to 92	
**Age groups**	< 25	511	10.9
(years)	25 - 34	620	13.4
	35 - 44	895	19.3
	45 - 54	811	17.5
	55 - 64	734	15.8
	65 - 74	718	15.5
	> 74	351	7.6
**Marital status**	married, living together	2,470	53.2
	married, living separately	56	1.2
	Single	1,127	24.3
	Divorced	444	9.6
	Widowed	543	11.7
**Education**	not graduated	49	1.1
	Pupil	164	3.5
	8^th^ grade (Hauptschule)	2,018	43.5
	10^th^ grade (Mittlere Reife/Realschule/POS)	1,622	34.9
	technical school	135	2.9
	12^th^/13^th^ grade (Abitur)	357	7.7
	university/college degree	295	6.4
**Employment status**	full-time (≥ 35 hours)	1,689	36.4
	part-time (15–34 hours)	402	8.7
	part-time (≤14 hours)	89	1.9
	Unemployed	252	5.4
	Pensioner	1,385	29.9
	unable to work	427	9.2
	in professional training	59	1.2
	in school-/college education	337	7.3
**Household net income**^**#**^	< 750 € per month	177	3.8
(*N* = 4,413; 95.1%)	750 to 1,250 € per month	793	17.1
	1,250 to 2,000 € per month	1,584	34.1
	> 2,000 € per month	1,858	40.1

Note. ^#^ percentages are representing the whole sample with N = 4,640.

### Measures

In the new ultra-short screening version (FEE-US), participants rate all 12 items, i.e. six for the mother and six for the father, on a four-point Likert scale in respect to how often they have experienced a certain situation in their childhood (1 = *No, never*, 2 = *yes, occasionally*, 3 = *yes, often*, 4 = *yes, always*). In the ultra-short screening version investigated here (FEE-US), each of the three scales consists of two items for mother and father, respectively. Scale values of the three scales for each mother and father are calculated by simply adding the value of each assigned item, resulting in a range of 2 to 8 for the FEE-US.

### Statistical procedure

Analyzed data format was binary for gender, categorical for the FEE items as well as age groups, and continuous for the latent variables (factors, scales). Item analyses of the FEE data were carried out using SPSS 16.0 and PRELIS 2.80s software. Confirmatory factor analyses were done using Mplus 6.12. Item response theory (IRT) parameter estimation used R 2.14.2 software with the eRm 0.14-0 library package. The following procedure has recently been shown to be useful in the reevaluation and refinement of existing instruments (e.g. [21]).

First, the respective empirical model fit of the hypothesized three-factor model was tested. The significant non-normality of categorical data as indicated by item analyses was taken into account by applying the Robust Weighted Least Square estimation (WLSMV) with a mean- and variance adjusted chi-square test statistic that uses a full weight matrix (see [22] pp. 399–400). For the evaluation of the model fits, the following thresholds appeared to be appropriate: good model fit is indicated by a Comparative Fit Index (CFI) as well as a Tucker-Lewis-Index (TLI) above .95 [23], and a Root-Mean-Square Error of Approximation (RMSEA) of less than .05, while a RMSEA between .05 and .079 is considered to be adequate [24].

Second, it was tested whether the factor structure differed between men and women, as well as across age groups. Therefore, models with multiple indicators and multiple causes (MIMIC models) were calculated. In each calculation, the respective covariate was regressed both onto all latent variables and all onto indicators. Given an at least acceptable model fit, a significant association between covariate and latent variable indicates group differences in latent means, i.e. population heterogeneity. A significant association - that is also meaningful as indicated by a standardized loading above .20 (see [25], pp. 268, 304–316) - between co-variable and indicator points toward measurement invariance, i.e. differential item functioning (see [25], p. 268). Indicators at risk for measurement invariance were identified by a significant modification index above 3.84 in CFA models for father AND mother items, where the direct effect of the covariate on each indicator was fixed to zero. However, due to the little number of observed variables, no simultaneous exploratory modeling could be done for latent variables and indicators. Thus, only indicators and the related concept of measurement invariance were examined in the MIMIC framework. Subsequent MANOVAs tested for differences in means of observed raw score sums across two independent variables (gender, age group) and six dependent measures (factors 1 to 3 for both parents). MANOVA effect sizes of η^2^ > .01 are considered to be weak, of η^2^ > .09 to be moderate, and η^2^ > .20 to be strong according to Cohen (see [26], p. 268).

Third, items and factors were tested in an IRT framework. How items actually map the dimension (latent trait), i.e. item discriminability, is represented by the obtained model-based thresholds.

Fourth, factors were examined for their discriminant and convergent factor validity as well as their relationship to raw values. Factor-based convergent validity is established when the average variance explained (AVE) by each factor is ≥ .50 [27]. Factor-based discriminant validity is proven if the square root of the respective AVE is above the correlations with any other related factors in the model [28]. The usefulness of raw scores in ranking respondents along the overall latent trait was explored through correlations between the Mplus-generated factor scores, and the respective total raw scores.

## Results

### Descriptive item analysis

As seen in Table 2, items appraisal differed between the scales, regardless of the mother or father format. Significant univariate non-normality was found through the Shapiro-Wilk test with all *W* > .34 (all *p* < .001), as well as for both skewness and kurtosis with one exception on scale level (Control & overprotection of the mother) and two on item level (items 11 for mother and father). Most items and scales tended to be significantly left-skewed and spikier than the Gaussian distribution. Significant multivariate non-normality according to Mardia [29] was found for father items with multivariate skewness, β_1,*p*_ = 11.08, *χ*^2^ = 8,565.51, *p* < .001, and multivariate kurtosis, β_2,*p*_ = 66.12, *n* (β_2,*p*_) = 63.0, *p* < .001, as well as for mother items with β_1,*p*_ = 17.19, *χ*^2^ = 13,295.29, *p* < .001 and β_2,*p*_ = 75.80, *n* (β_2,*p*_) = 96.6, *p* < .001.

**Table 2:** FEE-US item and scale characteristics

**Scale item**		***M (SD)***	**Response frequencies**			
			**Never**	**Occasionally**	**Often**	**Always**
*Father – Rejection & punishment (Factor 1)*		2.7 (1.06)				
	01 Have you been punished hard by your father, even for trifles (small offenses)?	1.5 (0.69)	59%	32%	8%	1%
	18 Did it happen that your father gave you corporal punishment without reason?	1.2 (0.53)	82%	14%	3%	1%
*Father - Emotional warmth (Factor 2)*		4.2 (1.49)				
	15 Has your father comforted you when you were sad?	2.2 (0.81)	20%	47%	27%	6%
	24 Was your father able to smooch with you?	2.0 (0.85)	29%	45%	20%	6%
*Father – Control & overprotection (Factor 3)*		3.3 (1.16)				
	11 Did your father spur you to become the best?	1.8 (0.83)	46%	35%	15%	4%
	23 Do you think that your father’s anxiety that something might happen to you was exaggerated?	1.5 (0.67)	58%	34%	7%	1%
*Mother – Rejection & punishment (Factor 1)*		2.5 (0.90)				
	01 Have you been punished hard by your mother, even for trifles (small offenses)?	1.4 (0.62)	70%	24%	5%	1%
	18 Did it happen that your mother gave you corporal punishment without reason?	1.1 (0.44)	89%	8%	2%	1%
*Mother - Emotional Warmth (Factor 2)*		5.3 (1.42)				
	15 Has your mother comforted you when you were sad?	2.7 (0.77)	6%	31%	50%	13%
	24 Was your mother able to smooch with you?	2.6 (0.83)	9%	34%	44%	13%
*Mother - Control & overprotection (Factor 3)*		3.6 (1.29)				
	11 Did your mother spur you to become the best?	1.7 (0.79)	49%	35%	13%	3%
	23 Do you think that your mother’s anxiety that something might happen to you was exaggerated?	1.9 (0.84)	39%	39%	18%	4%

Note. N = 4,640. Item numbering according to Schumacher et al. [1,2], item translations by the authors according to Arrindell et al. [9]. Mean range is 1 - 4, with lower/ higher scores indicating refusal / stronger approval. Non-normality tests of skewness and kurtosis: *** p < .001, ** p < .01. W = Shapiro-Wilk test of normality.

### Confirmatory factor analysis (CFA)

Item assignments of the hypothesized three factor model were adopted from the original FEE [1,10] and can be seen in Table 2. Each of the three factors loads on two observed indicator variables. All factors and indicators are present twice, once concerning the mother and once concerning the father of the participant. As a prerequisite for analyses in Mplus 6.12, the item with the highest loading on the respective factor in an exploratory factor analysis using Principal Axis extraction (not reported here) was chosen to be the factor’s marker indicator, i.e. their factor loading being fixed to one. Errors were specified as random and uncorrelated. Detailed analysis concerning modification indices (not reported here) suggested further relaxation of model specifications, i.e. freely estimated correlations between items 11 and 13, respectively, with all other items. However, this resulted in unidentified models due to the relatively little number of observed variables compared to estimated parameters. Furthermore, there is no theoretical assumption on which theses relaxations could have been based. Thus, no such relaxation could be modeled.

The overall fit statistics as shown in Table 3 suggest an adequate fit to the empirical data as indicated by good CFI values, good or adequate TLI values, and adequate to inadequate RMSEA 90% CI upper bound values. The nested *χ*^2^ difference test resulted in significant differences of *χ*^2^-values for the mother model (χ^2^_diff_ (2) = 31.69, *p* < .001), however no significant differences were found for the father model (χ^2^_diff_ (2) = 4.00, *p* = .136). Estimated factor loadings were significantly related to their respective latent factors with *r*^2^ between .18 and .72, all *p* < .001. Standardized factor intercorrelations were small to moderate (see Table 4).

**Table 3:** Confirmatory factor analysis: model fit (Robust Weighted Least Square estimation)

**Model tested**	**Absolute fit**		**Comparative fit**		**Parsimony fit**
	***χ***^**2**^^**a**^	***df***	**TLI**	**CFI**	**RMSEA (90% CI)**
*Factorial Invariance*					
Father items	136.380	6	.968	.987	.068 (.059, .079)
Mother items	235.740 ***	6	.933	.973	.091 (.081, .101)
*MIMIC models – gender*					
Father items – exploratory	241.923	9	.948	.978	.075 (.067, .083)
Father items – constrained ^b^	344.823	10	.933	.968	.085 (.077, .093)
Mother items – exploratory	268.581	9	.931	.970	.079 (.071, .087)
Mother items – constrained ^b^	285.301	10	.934	.968	.077 (.069, .085)
*MIMIC models – age group*					
Father items – exploratory	205.642	9	.956	.981	.069 (.061, .077)
Father items – constrained ^b^	314.059	8	.923	.970	.091 (.082, .100)
Mother items – exploratory	331.585	9	.913	.963	.088 (.080, .096)
Mother items – constrained ^b^	347.002	8	.897	.961	.096 (.087, .104)

Note. N = 4,640. ^a^ mean- and variance-adjusted X^2^-test statistic that uses a full weight matrix. The chi-square difference test for nested models is described in [BR06], pp. 394-396. ^b^ see methods section for further details on MIMIC modeling. TLI = Tucker-Lewis index. CFI = Comparative fit index. RMSEA = a Root-Mean-Square Error of Approximation. 90% CI = 90% confidence interval.

**Table 4:** Confirmatory factor analysis: Factor matrix

	**Father items**				**Mother items**			
	**Factor 1**^**a**^	**Factor 2**^**a**^	**Factor 3**^**a**^	**δ**_***i***_	**Factor 1**^**a**^	**Factor 2**^**a**^	**Factor 3**^**a**^	**δ**_***i***_
	***Λ***_***i*(1)**_	***Λ***_***i*(2)**_	***Λ***_***i*(3)**_		***Λ***_***i*(1)**_	***Λ***_***i*(2)**_	***Λ***_***i*(3)**_	
i1	.83 (.80, .86)			.31	.70 (.66, .74)			.51
i18	.85 (.82, .89)			.27	.93 (.88, .98)			.14
i15		.86 (.82, .89)		.27		.84 (.82, .87)		.29
i24		.81 (.78, .84)		.34		.79 (.76, .82)		.38
i11			.54 (.49, .59)	.71			.54 (.50, .58)	.71
i23			.47 (.42, .51)	.78			.58 (.54, .62)	.66
f1 ⇔ f2 ^**b**^		-.41 (−.44, -.38)				-.39 (−.43, -.35)		
f1 ⇔ f3 ^**b**^		.59 (.53, .65)				.48 (.42, .54)		
f2 ⇔ f3 ^**b**^		.37 (.31, .41)				.49 (.44, .53)		

*Note*. *N* = 4,640. Item enumeration according to Schumacher et al. [1,2], see also Table 2. The robust weighted least square estimation was used; δi = Measurement error (uniqueness), i.e. residual variances of completely standardized parameters. ^a^ For item-factor loadings, only completely standardized loadings are reported, i.e. completely standardized regression weights, with 95% confidence intervals in brackets. ^b^ For factor correlations, 95% confidence intervals are presented in brackets.

### Effects of gender and age

Multivariate influences on the three-factor structure were examined for gender and age group. As a prerequisite for such analysis, model fit as judged by CFI and RMSEA was acceptable for most of the MIMIC models (see Table 3). While certain items qualified for possible differential item functioning both for gender and age group, no significant and considerable effects on items were found with standardized loadings *λ*_STD_ < .13, all *p* < .05. Thus, it seems likely that each item is interpreted equally by the respective groups. Multivariate analyses of variance for observed raw score sums resulted at best in significant, but small effects for age with *F*_*Wilks-Lambda*_(36, 20295) = 7.46, *p* < .001, η^2^_part_ = .010 and for gender with *F*_*Wilks-Lambda*_(6, 4621) = 28.19, *p* < .001, η^2^_part_ = .035. For, the MANOVA results are similar for age with *F*_*Wilks-Lambda*_(36, 20295) = 6.79, *p* < .001, η^2^_part_ = .009 and for gender with *F*_*Wilks-Lambda*_(6, 4621) = 28.72, *p* < .001, η^2^_part_ = .036. Older participants reported more rejection & punishment, less emotional warmth, and less control & overprotection equally for both parents with all *F*(6, 4633) = 4.33 to 18.42, all *p <* .001, all η^2^_part_ = .006 to .022. Females reported more emotional warmth from both parents and less rejection & punishment from the father with all *F*(1, 4638) = 39.07 to 106.50, all *p <* .001, all η^2^_part_ = .008 to .023. Since MANOVA results for raw scores and factor scores are identical in yielding only small effects, population heterogeneity might be a given if analyzed in a CFA MIMIC model.

Effects of parental gender are not evident in two of the three scales with mother and father seeming to be nearly equally often rejecting, punishing, controlling, and overprotecting. Notably, the absence of paternal emotional warmth as shown in Table 2 with 20% and 29%, was three times more often reported than absence of maternal emotional warmth with 6% and 9%.

### Parameters in the IRT framework

**Figure 1:**
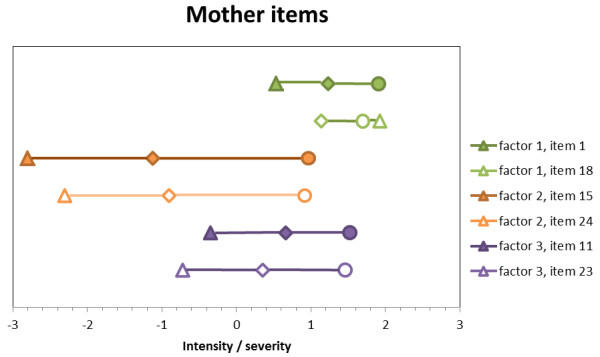
**Mother items’ thresholds pertaining to the four-level continuous item. **Note: Threshold 1 = triangle. Threshold 2 = rhombus. Threshold 3 = circle.

**Figure 2:**
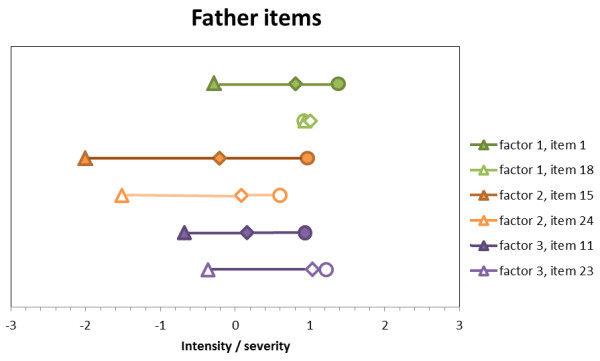
**Father items’ thresholds pertaining to the four-level continuous item.** Note: Threshold 1 = triangle. Threshold 2 = rhombus. Threshold 3 = circle.

The approximately thresholds for each item are displayed in Figures 1 and 2. As required in IRT, items differ in their position on the latent dimension and produce thresholds as expected except for item 18 both in its mother and father format. Notably, items do not cover the full spectrum. For factor one, mother items and father items cover 23% and 28%, respectively. Factor two has a better coverage with 63% and 50%, respectively, while factor three is not covered well with 37% and 32%, respectively.

### Factor validity and relationship to raw scores

AVEs, inter-factor correlations, as well as correlations between factor scores and raw scores can be seen in Table 5. Convergent factor-based validity holds for all factors. Discriminant factor-based validity is indicated for all factors by AVE square roots exceeding the factor intercorrelations. Factor scores are moderately intercorrelated. The high correlations between the specific raw scores and the respective factor-based scores with *r*_ij_ = .78 to .99, all *p* < .001 substantiate that scale sums are well-suited to rank respondents along the latent dimension.

**Table 5:** Average variance extracted (AVE) and correlation matrix between factor-based scores and raw scores

			**Factor score**			**Raw score**		
	**AVE**	**√AVE**	***f1***	***f2***	***f3***	***f1***	***f2***	***f3***
**Father items**								
**Factor score**								
*f1*	.97	.98	1.00					
*f2*	.96	.98	-.49***	1.00				
*f3*	.51	.71	.57***	.43***	1.00			
**Raw score**								
*f1*			.93***			1.00		
*f2*			-.44***	.99***		-.49***	1.00	
*f3*			.42***	.24***	.78***	.57***	.43***	1.00
**Mother items**								
**Factor score**								
*f1*	.93	.97	1.00					
*f2*	.95	.98	-.47***	1.00				
*f3*	.62	.79	.42***	.59***	1.00			
**Raw score**								
*f1*			.86***			1.00		
*f2*			-.45***	.99***		-.47***	1.00	
*f3*			.41***	.35***	.85***	.42***	.59***	1.00

Note. N = 4,640. All AVEs calculated from completely standardized indicator loadings of the respective model.

## Discussion and conclusion

The aim of the present study was to evaluate the factorial structure and item properties of an ultra-short screening version of the FEE questionnaire by means of a confirmatory factor analysis using a representative, cross-sectional German sample. Furthermore, sociodemographic influences on the perceived parental rearing behavior were investigated.

The results of the CFAs showed a generally good fit for the three-factor model, both for father and mother items. This confirmed the a-priori hypothesized item assignment which was adopted from German EMBU translation (FEE, see [1,2]. Therefore, the recalled parental rearing behavior of the mother and the father could be described along three dimensions: rejection & punishment, emotional warmth and control & overprotection. These three scales for each parent that are distinct and can adequately depict recalled parental rearing behavior.

The three dimensions are stable across genders and across age groups, indicating that the instrument comprises of the same factors when applied to men or women at the age of 14 to 92. Raw score sums were demonstrated to be reliable estimators of the actual recalled rearing behavior. However, it has to be noted that in an IRT framework, the latent factors are not fully covered by the chosen items which might be the consequence of non-normal distributed data. Choosing other items of the original scales as indicators could solve this shortcoming. Nevertheless should it be possible to order individuals, or more precisely their memories, along that dimension. By providing population-based norms, future studies might provide necessary information for individual assessments so that individuals can be compared to their respective cohort. Notably, no estimations of scale reliability or internal consistency are reported since at least four items per factor or scale have to be present in order to calculate scale reliabilities and corrected item-scale correlations [30]. This shortcoming was inevitable as scales comprised of only two items, leading to awkward values that do no longer represent meaningful scale characteristics. On the other hand, shortening instruments as far as possible is a common sense goal when constructing short forms of existing instruments.

Whereas based on the EMBU only a few studies reported effects of gender [31] and age [8,32], the presented data indicated some significant but small influences of these factors on recalled parental rearing behavior. Previous findings from the FEE authors indicate similar effects [2]. Based on their memories, older subjects recalled their parents as more rejecting and less emotionally warm than did their younger counterparts. So, the older subjects do not idealize the parental rearing behavior more than the younger subjects did [8,32]. This age effect might be explained by historical changes in parenting attitudes and behavior in child rearing practices of the investigated German sample. This is a possible change from Prussian values like discipline and order in the parents of the old cohort to more child-centered rearing practices in the post-modern information society with its diversity and wealth of information on “good” parenting. This can only be understood when reflecting the specific historical background in each cohort. For example, the now 70 to 80 year olds grew up during and shortly after the Second World War, suffering from dissipation, dead or disabled fathers, malnutrition, overcharged mothers and so on. In contrast, the next generation which was born and raised in the 1950s and 60s grew up in rapidly increasing material and economical wealth during the German “economic miracle” in addition to increasingly liberal society after the student uprisings in 1968 throughout Europe. In turn, these events might change values underlying and conditions surrounding child rearing, however, this is still to be proven for children from different socio-economical backgrounds.

Furthermore, based on their memories, the female subjects reported having received more emotional warmth from their fathers than the male subjects did, whereby the latter recalled their fathers as stricter and more rejecting. These differences might be explicable by the urge of the father to rear their son stereotypically, i.e. to be strong and silent.

These sociodemographic tendencies have to be considered for the implementation of the 12-item FEE-US in large prevention studies. Therefore, norm values as well as cut off values for FEE-US would be of help for an implementation in the prevention field. However, these considerations are limited to the point that only one cohort from one country is examined, leaving room for speculation on the variability and cultural specificity of child raising behavior (e.g. [33]).

The retrospective assessment of recalled parental rearing behavior represents a specific problem in assessing the actual parental rearing experienced during childhood or its subjective representation [34,35]. The subjective representation may reflect the present mood, errors in autobiographical memory (un-/conscious distortions), false memories or idiosyncratic reconstructions of the subjects’ personal history. However, the existing literature did not provide consistent and conclusive data on the mood-congruent recall of relevant personal stimuli [33,34,36-38] as well as on the validity of retrospective data on parental rearing behavior [39]. Therefore, longitudinal studies with independent raters that are outside of family as observer should be considered for the validity of parental rearing practices [40]. Unfortunately, in clinical practice, the child rearing experienced by patients can only be assessed retrospectively after the onset of the disorder. Nevertheless, the then obtained information can be of help in the therapeutic process.

The strength of this study is the large representative sample and the statistical approach of these results. However, large sample size could easily lead to small but significant correlation coefficients, which is underlined by the small effect sizes found. In addition, the capacity of each scale to capture the continuum of the underlying dimensions is limited with these 2-item-per-factor scales. The FEE-US is only a screening instrument and additional assessments on recalled parental rearing, i.e. by the long version or interviews, are necessary for more profound conclusions. Herefore, further studies are recommended for referencing the FEE-US to standard instruments/procedures (e.g., the long FEE version or observations in a longitudinal study) in order to assess how many false negative and false positive cases would be involved. This insight would be useful both in a clinical and a research framework.

In summary, the present data show that even in an ultra-short screening version of the EMBU and its German version, FEE, the factorial structure could be replicated, which reflects the quality of the instrument for the retrospective assessment of subjective representations of parental rearing behavior. The resulting FEE-US screening instrument enables to screen in time-saving manner for recalled parental rearing behavior as a risk factors for the development of mental disorders in large samples. Next to this factor, other possible risk factors or aspects can be assessed simultaneously and still keep the survey applicable. Moreover, this information will be relevant not only to the research concerning psychological disorders but also to the field of non-clinical applications such as prevention projects and counseling settings. Based on an early screening, the detection of such a risk factor might lead to supporting parents in finding a more positive rearing style. After the screening, intervention programs can be implemented more precisely to the population in need and possible chronifications of diseases and their cost-expensive treatment can be avoided.

## Competing interests

The authors declare that they have no competing interests.

## Authors' contributions

EB was responsible for the conception and the design of the study as well as the acquisition of the data. SP performed the statistical analysis. SP, KP, and MZ contributed to the interpretation of the data. KP and SP wrote the first and final version of the manuscript, and critically revised the manuscript for intellectual content. All the authors read and approved the final version of the manuscript for publication.

## Pre-publication history

The pre-publication history for this paper can be accessed here:

http://www.biomedcentral.com/1471-2288/12/169/prepub
